# One-minute sit-to-stand test as a quick functional test for people with COPD in general practice

**DOI:** 10.1038/s41533-023-00335-w

**Published:** 2023-03-15

**Authors:** J. G. Spence, J. Brincks, A. Løkke, L. Neustrup, E. B. Østergaard

**Affiliations:** 1 Department of physio- and occupational therapy at Aarhus University Hospital, Aarhus, Denmark; 2 Research Centre for Health and Welfare Technology - Programme for Rehabilitation, VIA University College, Aarhus N, Denmark; 3 Research Centre for Health and Welfare Technology – Programme for Mind and Body in Mental Health, VIA University College, Aarhus N, Denmark; 4 Department of Medicine, Little Belt Hospital, Vejle, Denmark; 5 Department of Regional Health Research, Faculty of Health Scienties, The University of Southern Denmark, Odense, Denmark; 6 General Practice, Vejle, Denmark

**Keywords:** Health services, Rehabilitation, Lifestyle modification

## Abstract

Assessing changes in functional exercise capacity is highly relevant in the treatment of people with Chronic Obstructive Pulmonary Disease (COPD), as lung function is often static. In Denmark, most people with COPD are followed in general practice where traditional functional tests, like six-minute walk test, require too much time and space. Therefore, there is an urgent need for a quick functional exercise capacity test that can be performed in a limited setting, such as general practice. This study aimed to identify a quick test to measure functional exercise capacity in people with COPD and identify which factors could affect the implementation of such a test in general practice. A mixed method feasibility study composed of a literature review and qualitative interviews was used. Quick functional tests for people with COPD were identified and evaluated through the COSMIN methodology. For the interviews, 64 general practices were included, and 50 staff members and 14 general practitioners (GPs) participated in the interviews. Responses were categorized and thematically analyzed. The 1 min sit-to-stand-test (1 M STST) was found suitable for a general practice setting. The COSMIN methodology rated it “sufficient” in reliability (ICC 0.90–0.99), measurement error (MID 2.5–3), construct validity and responsiveness (AUC 0.72), and found a moderate to strong correlation in criterion validity (*r* = 0.4–0.75). Several GPs wished for a quick functional test and emphasized evidence, information, and limitations as essential when deciding on implementation. Other factors identified included time, other tests, and economy. 1 M STST is a valid test to assess functional exercise capacity in people with COPD. The test is quick and can easily be performed in a standard consultation, and several GPs wished for such a test.

## Introduction

In Denmark, most people with Chronic Obstructive Pulmonary Disease (COPD) are treated in general practice and assessed at annual check-ups^1,2^. However, despite strong evidence for the positive impact of physical activity and rehabilitation, for people with COPD^3,4^, studies have uncovered a significant focus on medication and only a minor focus on physical activity among general practitioners (GPs) in Denmark^5,6^.

As a part of pulmonary rehabilitation, healthcare professionals measure improvements in functional exercise capacity^5^. This measurement is essential as people with COPD have a significantly lower activity level than healthy, age-matched individuals^7^. There are many barriers to physical activity, such as lack of motivation, fear of shortness of breath or anxiety^5,8^. Therefore, assessing changes in functional exercise capacity is vital to ensure that people with COPD stay physically active.

Functional exercise capacity can be described as a persons maximal performance in the physical domain^9,10^. If functional exercise capacity decreases, symptoms will likely increase as the functional reserve during everyday activities will diminish. In COPD, reduced functional exercise capacity may lead to increased dyspnea and fatigue, which can trigger anxiety during certain activities and therefore cause inactivity. Exercise and increased physical activity could prevent this vicious circle by improving functional exercise capacity and decreasing symptom burden^3^.

The lung function test (spirometry) is often used in general practice to diagnose COPD and to assess the degree of lung function impairment^2^, and for this purpose, the test is valid^11^. However, lung function does not correspond to functional exercise capacity^7^. Because of the progressive nature of COPD, lung function will often decrease over time, even if functional exercise capacity improves. This might negatively impact the motivation for continued physical activity and reduce the GP’s incentive for positive dialogue about physical activity^6^.

The Global strategy of management, diagnosis and prevention of COPD describes important guidelines and mentions functional exercise capacity as one of the important factors in describing disease severity and progression of COPD^11^. The guidelines recommend the 6 min walking test (6MWT) for testing functional exercise capacity at check-ups in general practice^11^. Unfortunately, with limited time and space, traditional tests are difficult to implement in general practice. Therefore, there is an unmet need for a quick and feasible functional exercise capacity test—especially in general practice.

Our study aimed to (1) identifying a quick, valid functional test capable of assessing functional exercise capacity for people with COPD in settings where time and space are limited and (2) exploring factors, through interviews with staff members and GPs, that could affect the implementation of such a test in general practice.

## Methods

A feasibility study with method triangulation was conducted. The study consisted of a literature review and fieldwork interviews with 64 general practices in Denmark in 2021. The feasibility design was based on two framing questions: Does it work? Will it work?^12^. The framing questions made up the two phases of our study. They originated from a preconception of general practice to chronologically explore a hypothetical implementation of a quick functional test in general practice. First, a search for systematic reviews involving functional exercise capacity tests for people with COPD was conducted. Second, an assessment of the most suitable functional exercise capacity tests for limited settings was performed using the COSMIN methodology^13^. Qualitative, semi-structured interviews with 64 general practices were performed: 50 staff members (medical secretaries and nurses) were interviewed by telephone and 14 GPs by e-mail. Responses were categorized and thematically analyzed.

### Data collection—phase one

The search for literature was conducted within EMBASE, PUBMED and CINAHL. A PICOT - approach was used and focused on systematic reviews concerning functional tests for people with COPD (Supplementary Figure 1). After titles and abstracts were screened, it resulted in five systematic reviews^14–18^. Inclusion and exclusion criteria for the quick functional tests were conceived from the preconceptions of general practice. Functional exercise capacity tests were excluded if specialized equipment or skills unsuitable for GPs or nurses were required, if a space larger than 6 × 4 meters was required, or if the time required for the test was 5 min or more (including instruction). For inclusion, the test had to be valid to assess functional exercise capacity for people with COPD and comparable with traditional functional tests like the 6MWT and the Shuttle walk test (SWT).

Based on the criteria and literature about functional tests, the 1 min sit-to-stand-test (1 M STST) was found most suitable to measure relevant clinical outcomes in people with COPD^19,20^. A PICO-search was performed within the before-mentioned databases focusing on validity and correlation with functional tests such as 6MWT and the SWT (Supplementary Figure 2). Three articles were found after abstracts were read^19,21,22^. The reference lists for eligible trials were screened for additional relevant articles, and four more were found^20,23–25^. In total, seven articles were included in the COSMIN assessment.

### COSMIN assessment

The 1 M STST was assessed using the COSMIN methodology. The COSMIN methodology is a modular tool to review outcome measurement tools systematically. The COSMIN Checklist was chosen as it specifically reviews the measurement tool and its properties by using the articles, instead of evaluating the articles independently. The version of the COSMIN checklist was created for patient-reported outcomes measurements, but it is also recommended for performance-based measurements^13^.

The checklist consists of four stages: (1) The measurement properties (Validity, reliability etc.) from the 1 M STST, which were investigated in the included articles, were identified. (2) The included articles’ methodological quality was evaluated using the *COSMIN Risk of Bias checklist*. (3) The investigated measurement properties and their outcomes from the included studies were evaluated through the standards of good measurement properties from the *COSMIN criteria for good quality*. (4) The evidence was then summarized, and the quality of the summarized evidence was evaluated with the *GRADE approach* from the COSMIN methodology^13,26^.

The results for each measurement property were accumulated and assessed as *sufficient, insufficient, inconsistent* or *indeterminate*. The quality of evidence in each measurement property was evaluated based on studies investigating the measurement property in the following areas: Risk of bias, inconsistency, imprecision and indirectness^13^.

### Data collection—phase two

Literature and documents about general practice in Denmark were explored on government and general practice relevant websites, focusing on annual check-ups and monitoring, organizational structure and economy in preparation for composing relevant questions for the interview^2,27–29^. Two interview guides were composed, one with a general focus and one with a specific focus (Supplementary Figure 3). The general focus interview addressed staff members performing tests and time allocated for testing and was conducted as telephone interviews with 50 staff members from general practice. The specific focus interview addressed staff, time, tests performed at annual check-ups, and factors regarding implementing a quick functional test for people with COPD and was conducted as e-mail interviews with 14 GPs. Telephone and e-mail interviews were used because of the COVID-19 pandemic during the testing period.

The results were collected and arranged. One of the staff members and two of the GPs, could not answer the question regarding time spent. Furthermore, one GP did not elaborate on the tests for annual check-ups.

In questions where an interviewee had two answers in the same category, e.g., if both nurses and doctors were conducting the tests or more than one test were performed during the consultation, both answers were included. Still, the total number of answers was not increased.

### Data analysis

Answers about factors regarding implementing a quick functional test were categorized and thematically analyzed. For the analysis of the empirical data, Malterud’s thematic analysis was used^30^. This method was used because it identifies patterns and themes in the answers from the interviewees. The method uses decontextualization and recontextualization in a four-step approach to identify important themes in the qualitative datasets collected in the interviews. The steps were: An overall impression of the answers and themes in the interview, identification of meaningful entities in the answers, condensation of the entities and at last, a synthesis of the identified themes.

### Reporting summary

Further information on research design is available in the Nature Research Reporting Summary linked to this article.

## Ethics

The participants were informed about the aims of the study, the method and professional confidentiality. Based on that, the participants gave informed consent^31^.

The data was anonymized, including the names of the participants and the general practices to protect individual confidentiality according to The Declaration of Helsinki^31,32^. According to Danish law, Scientific Ethical Committees Act §14 no. 2, research based on interviews and questionnaires is exempt from ethical approval^33^. Since the study was solely based on interviews, ethical approval was not required by regulatory authorities in Denmark^33^.

## Results—phase one

As shown in Table 1, the 1 M STST has an overall rating from the COSMIN checklist of *sufficient* in reliability, measurement error, construct validity and responsiveness. The quality of evidence in these categories is rated high, as the studies included have a sample size above 100, show consistent results in multiple studies, construct validity, and share a similar confirmed hypothesis. The results in criterion validity are *inconsistent* as some of the values for correlation are below the criteria for good measurement qualities in the COSMIN methodology (*r* ≥ 70 or area under curve ≥0.70). This downgrades the quality of evidence from high to moderate as the results are inconsistent. However, the correlation is still rated as moderate to strong.

**Table 1:** COSMIN summary of evidence.

	Results	Overall rating	Quality of evidence
Reliability	ICC^a^-range: 0.902–0.99 Consistent results: yes Sample size range: 42–203	Sufficient	High: Multiple studies showing good effect
Measurement error	MID^b^-range: 2.5–3 Sensitivity 80% Specificity: 60%	Sufficient	High: One study with sample size *n* = 50–100+
Criterion validity	Correlation range: 0.4–0.75 AUC^c^: 0.82	Inconsistent	Moderate: Two out of four studies with correlation under COSMIN limit.
Construct validity	Hypothesis confirmed: 4/4	Sufficient	High: Similar hypothesis in correlation with 6MWT^d^, consistent results
Responsiveness	AUC: 0.716 SMD^e^: 0.87–0.91	Sufficient	High: Two studies with consistent results

^a^Intraclass Correlation Coefficient.

^b^Minimal Important Difference.

^c^Area Under Curve.

^d^Six minute walk test.

^e^Standard Mean Deviation.

## Results—phase two

Results from the general and specific interviews can be seen in Table 2.

**Table 2:** Answers from interviews.

Who are testing? (*n* = 64)	Nurses: 55 (85.9%)
	GP^a^ secretary: 3 (4.6%)
	Medical students: 9 (14%)
	Care-assistants: 5 (7.8%)
	Practice assistants: 3 (4.6%)
	GP’s: 3 (4.6%)
	Medical laboratory Technician: 1 (1.5%)
Amount of time allocated to testing at annual check-ups (*n* = 61)	10 min: 1
	15 min: 9
	20 min: 3
	15–30 min: 5
	25 min: 3
	30 min: 38
	40 min: 1
	45 min: 1
Which tests are performed? (*n* = 13)	Spirometry w/o reversibility: 13
	Electrocardiogram: 4
	Blood samples: 6
	KRAM^b^: 5
	CAT^c^-score: 4
	MRC-scale^d^: 3
	SpO2: 3
	Weight measuring: 3
	GOLD^e^-classification: 2
	Bloodpressure: 2
	Inhalation technique: 2

^a^General Practitioner.

^b^Diet – Smoking - Alcohol-consumption - Physical activity.

^c^COPD Assessment Test.

^d^Medical Research Council – Dyspnea scale.

^e^Severity of COPD.

From the general interview, it was found that most general practices (85.9%) used nurses or a mix of nurses and other staff members to perform tests at the annual check-up. Medical students were the second largest group to perform the tests in general practices (14%).

In most general practices, the regular amount of time allocated for the annual check-up was 30 min, with 38 practices having this as their fixed amount of time for tests to be performed. Nine practices allocated 15 min, and five allocated 15–30 min depending on the number of tests planned at the check-up.

All practices included in the specific interview (*n* = 13) performed spirometry with or without reversibility. Spirometry with reversibility was only performed at the first meeting in the included general practices. Otherwise, spirometry without reversibility was used. Other tests were blood samples (*n* = 6) and questions about diets, smoking status, alcohol consumption and physical activity level (In Denmark known as KRAM^34^) (*n* = 5).

The thematic analysis identified four themes important for the implementation of a quick functional test such as the 1 M STST: (1) Lack of evidence for a quick functional test, (2) meaningful information about the test is needed, (3) the test is not relevant, and (4) limitations of the test.

All the statements are made by GPs working in General Practices in Denmark. Most GPs (10 of 14) had a positive attitude toward implementing a quick functional test in general practice. One of the GPs replied:

*“We often need (quick) tests for qualifying the functional status of the patient.”*

However, they found it important and central that the test should be experienced as meaningful for people with COPD, and they wanted evidence for the test to be valid to assess functional exercise capacity:

*“Yes, we would implement the test, if it was relevant for the patient”*

*“You could definitely do it [Implement the test (red.)], if there is evidence that it will add something useful regarding the functional exercise capacity”*

Among both sides, the positive toward implementation and the negative toward implementation, there were mentions about limitations regarding the 1 M STST in general practice:

*“[…] I do have some who wouldn’t be able to finish because of decreased general condition”*

*“As a solo general practitioner, I don’t share information in a team and document primarily for my own sake. You make an observation from the waiting room to the office, which you then use for assessing the MRC [MRC Dyspnea Scale (red.)]”*

One GP considered the 1 M STST not challenging enough for younger people with COPD and maybe too challenging for older people with COPD and decreased general condition. Another GP considered general observations for Medical Research Center Dyspnea scales (MRC-scale) or 30 s sit-to-stand test (30STST) sufficient for assessing functional exercise capacity. A third GP stated that some general practices solely implement tests recommended by their professional association.

## Discussion

1 M STST is assessed as *sufficient* for the measurement qualities; reliability, measurement error, construct validity and responsiveness. The quality of evidence is assessed high for the same measurement qualities.

Criterion validity is assessed as *inconsistent* with moderate quality of evidence, as two out of four studies had correlation values below the limit for good measurement qualities according to COSMIN (*r* ≥ 0.7)^13^. However, the correlation *r* = 0.4–0.75 in the studies is still moderate to strong^19–21,23^. Studies assessing criterion validity, e.g., validity compared to the 6MWT, underline that the 1 M STST is comparable with the 6MWT^21–24^. However, it is relevant to point out that the 6MWT and the 1 M STST assess functional exercise capacity in two different relevant functions of daily life, walking and sit-to-stand. Therefore, they can never be fully comparable.

Several studies in the COSMIN assessment did not include people in a weakened state or with musculoskeletal problems^19,21,24,35^. Therefore, these people must be assessed individually, which GPs also pointed out in the interviews as a limitation of the test.

The possible implementation of a quick functional test in general practice and some ways to comply with challenges on this matter were analyzed. A large variation in general practises was found, which underlines the importance of cooperating with the specific general practice to uncover the specific factors regarding an implementation. To determine factors influencing the implementation process, specific focus areas were explored from the feasibility concept^12^: practicality, expansion and demand.

The 1 M STST is considered practical as the only remedies required for carrying out the 1 M STST are a chair and a stopwatch (Supplementary Figure 4). The test protocol is accessible and easy to perform, even considering the registered diversity among the staff members performing tests in general practice^36^ (Table 2). In regards to expansion the study found that general practice has an average time allotted for annual check-ups of 30 min. The lung function test, used by all interviewed GP’s, varies in time, whether acceptable results are obtained quickly, but requires ~10 min to be performed^37^. The GPs interviewed defined the test battery used for annual check-ups in general practice as a modulated toolbox, adjustable and dependent on the person’s needs, more than a rigidly defined test battery. If so, it is reasonable to believe that the 1 M STST can be implemented. The results of this study show that the 1 M STST should be included in the test battery based on its relevance to people with COPD.

The general practices in Denmark receive a fixed annual fee per person with COPD^27–29^. This means that implementation will not alter the economic frame as it is fixed annually. In other countries, other financial systems might be in place, but the 1 M STST should be implementable based on its relevance to the person and its accessibility and low requirement for time and space.

Several of the interviewed GPs asked for a quick functional test, and the requested evidence has been determined in this study through the COSMIN methodology. In making test results more tangible and usable for the people and the GP, reference values will be appropriate. For example, a study from 2013 by Strassmann et al. with 6.926 healthy adults gave insight into average values for healthy individuals classified in age and gender^38^. These reference values and the minimal important difference (MID) of three repetitions for the 1 M STST, will be relevant and useful information for the person and the GP when implementing the test in general practice^25^.

The 1 M STST complies with all inclusion and exclusion criteria from the initial literature search. It was found that the 1 M STST is valid compared to the 6MWT, and the test results correlate with the quality of life and 2-year mortality^23,24,35,39^.

In general practice, the MRC-scale assesses the need for pulmonary rehabilitation or intensified focus on physical activity^2^. One of the interviewed GPs used it for categorizing functional exercise capacity. The MRC-scale is self-reported, and in 2014 Callens et al. found that one in four people with cardio-respiratory disorders over-or underestimated their actual functional exercise capacity on recall, especially people diagnosed with COPD^40^. Therefore, the MRC-scale is problematic when it comes to identifying the need for intervention regarding physical activity, and the 1 M STST can provide a more objective measure of functional exercise capacity for the GP. Neither the MRC-scale nor the 30STST assess functional exercise capacity in people with COPD as the 1 M STST^19,20^.

The articles found in our present study also conclude that the 1 M STST has high test-retest reliability (ICC 0.99 (95% CI 0.97–1)) and low learning effect (ICC 0.93 (95% CI 0.83–0.97)), which means that it only needs to be tested once to get a reliable result^20,22^. This underlines the relevance of the 1 M STST when assessing functional exercise capacity in time-limited settings. The 1 M STST responds to changes in functional exercise capacity and has a MID of three repetitions^22,25^. These results on the 1 M STST are supported in an extensive systematic review from 2019, where the 1 M STST is recommended, especially in settings where time and space are limited^36^. A recent study from 2022 also found that having a follow-up using the 1 M STST also had a clinically relevant benefit on functional status in people with COPD^41^.

The method triangulation in this study has strengthened the feasibility concept by exploring the research aims in different ways from different perspectives. The process of this project was evaluated continuously with the four quality criteria in qualitative projects^42^. The COSMIN methodology findings in this study are comparable to earlier studies, strengthening the external validity^14,15^.

The in- and exclusion criteria for the literature search were created to find a quick functional test accessible to all varieties of general practice settings. As they were based on a preconception, the 1 M STST was performed on GPs and other staff members of general practice at a symposium on COPD to examine if it was feasible in general practices. Based on this feed-back, it was concluded that the criteria for the literature research were sufficient to identify a possible, feasible test.

This article explored only objective focus areas from the feasibility concept (Practicality, expansion, demand). Another important focus area, “Acceptability”, about how the patient and the one performing the test experience the 1 M STST, has not been investigated in this study^12^. None of the included studies have mentioned this either. In an implementation of the 1 M STST into General Practice, the subjective experience of the patient is of paramount importance and should be investigated further in future studies. Most of the interviews were done in one region of Denmark. The e-mail interviews were done with GPs from different areas of Denmark. The similarities in the results justify a generalization of our results to general practices. The variety of staff included and the accessibility of the 1 M STST, compared with the similarities in our results, justifies a generalization of the findings to most general practice.

A possible consequence of the firm structure in the 14 e-mail interviews with the GPs might be that the area regarding factors for implementing a quick functional test has not been fully explored. Focus group interviews could have given a more in-depth view of the barriers and needs in an implementation process. Still, it is believed that this study uncovers variation among general practices concerning attitude towards the test and practicalities.

The results are limited to knowledge about annual check-ups in general practice usable for future research and feasibility studies in this area. Although based on the Danish healthcare system, the results of this study may apply to other healthcare systems internationally, especially regarding the validity and practicality of the 1 M STST and the need for a quick functional exercise capacity test in general practice.

In conclusion, according to COSMIN criteria, the 1 M STST is a valid, reliable, and responsive test to assess functional exercise capacity for people with COPD in general practice. Despite great variation in general practice, the 1 M STST is suitable for implementation because it requires a minimum of time and space for implementation, gives valuable information regarding functional exercise capacity and has therapeutic relevance for people with COPD, especially in general practice.

The results from this study indicate a need among GPs for a quick functional test for people with COPD. The GPs requested that the 1 M STST was valid for assessing functional exercise capacity and that the test was experienced as meaningful for people with COPD. In addition, the 1 M STST works well for factors such as time for consultations, other tests, and economy, which are important in the implementation of a quick functional exercise capacity test.

## Supplementary information

Reporting Summary

Supplementary Material

## Data Availability

Anonymised data that support the findings of this study will be made available from the corresponding author upon request.
