# Supplementary material for: One-minute sit-to-stand test as a quick functional test for people with COPD in general practice
Source: NPJ Prim Care Respir Med. 2023 Mar 15;33:11. doi: 10.1038/s41533-023-00335-w (PMC10015133; doi:10.1038/s41533-023-00335-w)
Supplement: Supplementary file 2 — Supplementary Material [file 41533_2023_335_MOESM2_ESM.pdf]

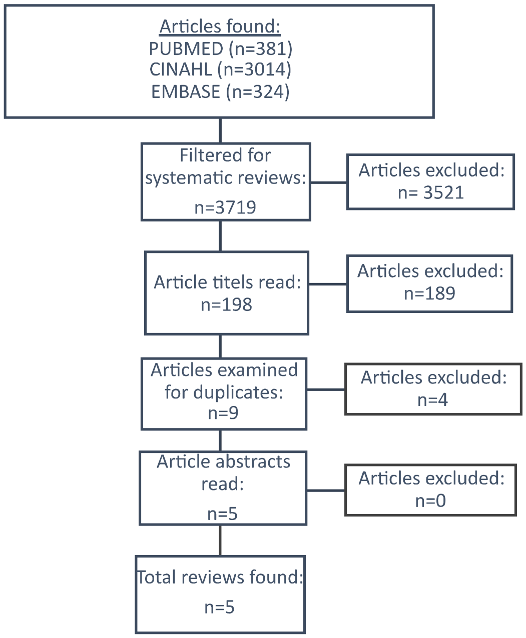
Articles found:
PUBMED (n=381)
CINAHL (n=3014)
EMBASE (n=324)
Filtered for systematic reviews:
n=3719
Articles excluded:
n= 3521
Article titels read:
n=198
Articles excluded:
n=189
Articles examined for duplicates:
n=9
Articles excluded:
n=4
Article abstracts read:
n=5
Articles excluded:
n=0
Total reviews found:
n=5

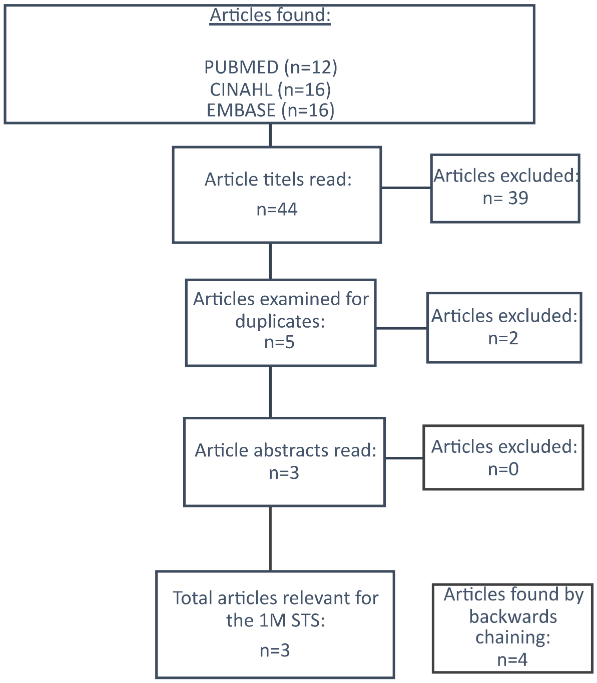
Articles found:
PUBMED (n=12)
CINAHL (n=16)
EMBASE (n=16)
Article titels read:
n=44
Articles excluded:
n= 39
Articles examined for duplicates:
n=5
Articles excluded:
n=2
Article abstracts read:
n=3
Articles excluded:
n=0
Total articles relevant for the 1M STS:
n=3
Articles found by backwards chaining:
n=4

Supplementary Figure 3

Interview questions

**General interview**<br>(Staff employees at General practices, by telephone)	**Specific interview**<br>(General Practitioners, by E-mail)
- Do you perform annual controls for people with COPD in your practice?<br><br>- Which employees perform the test during your controls with patients with COPD?<br><br>- How much time do you use for each annual control for people with COPD?	- Which employees perform the test during your annual controls with patients with COPD?<br><br>- How much time do you use for each annual control for people with COPD?<br><br>- Which tests do you perform during the annual control for people with COPD?<br><br>- Would you implement a quick functional capacity test (1-minute Sit-To-Stand test) in the annual control, if it was relevant for the person and their treatment?

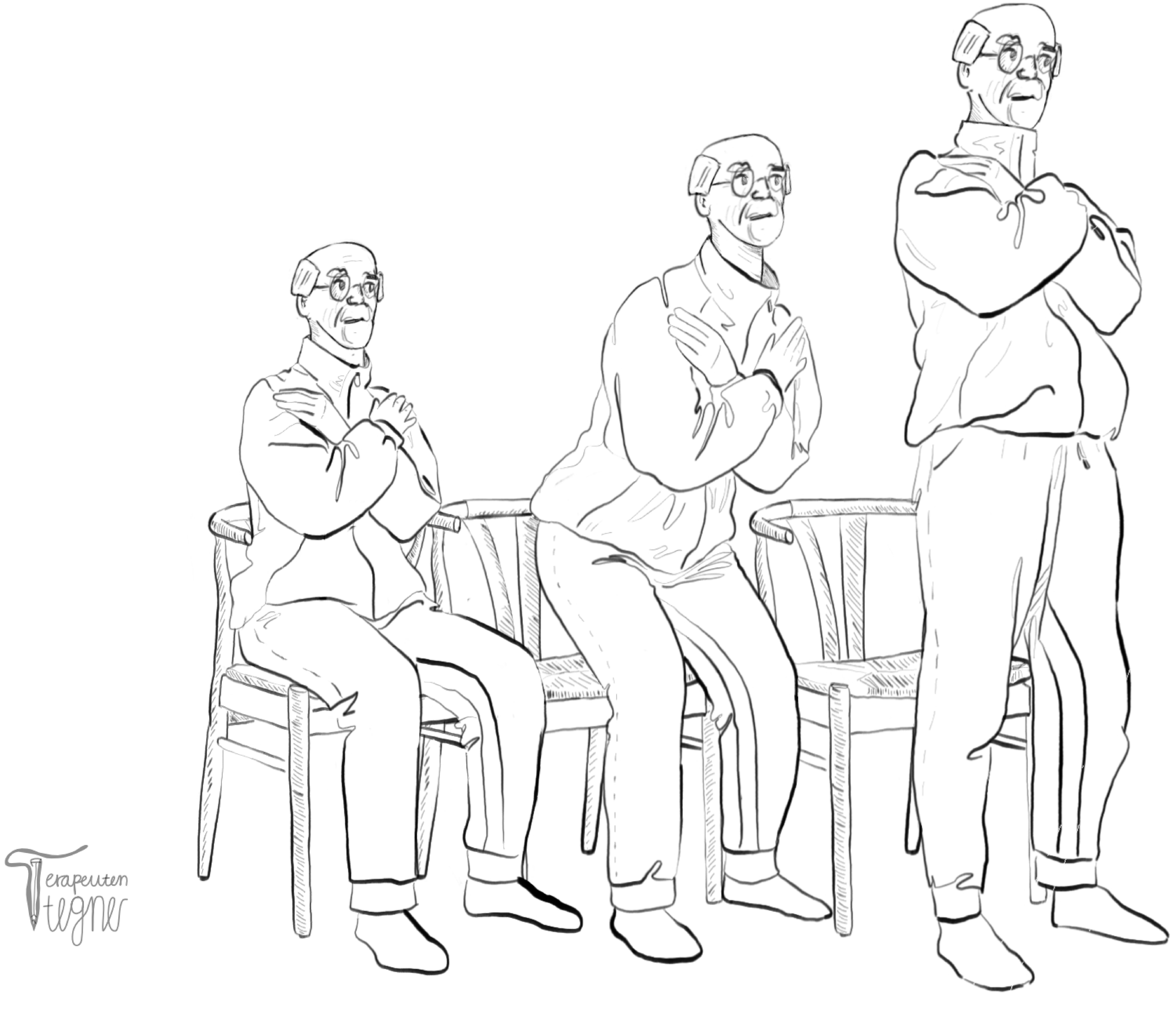
Terapeuten tegner
